# Spectral Characterization and 3D Molecular Modeling Studies of Metal Complexes Involving the O, N-Donor Environment of Quinazoline-4(3H)-one Schiff Base and Their Biological Studies

**DOI:** 10.1155/2014/817365

**Published:** 2014-02-11

**Authors:** Kuruba Siddappa, Sunilkumar B. Mane, Deene Manikprabhu

**Affiliations:** ^1^Department of Post-Graduate Studies and Research in Chemistry, Gulbarga University, Gulbarga, Karnataka 585 106, India; ^2^Department of Microbiology, Gulbarga University, Gulbarga, Karnataka 585 106, India

## Abstract

A simple condensation of 3-amino-2-methylquinazoline-4-one with 2-hydroxy-1-naphthaldehyde produced new tridentate ONO donor Schiff base ligand with efficient yield. The structural characterization of ligand and its Cu(II), Ni(II), Co(II), Mn(II), Zn(II), and Cd(II) complexes were achieved by the aid of elemental analysis, spectral characterization such as (UV-visible, IR, NMR, mass, and ESR), and magnetic data. The analytical and spectroscopic studies suggest the octahedral geometries of Cu(II), Co(II), Ni(II) and Mn(II) complexes and tetrahedral geometry of Zn(II) and Cd(II) complexes with the tridentate ONO Schiff base ligand. Furthermore, the conclusions drawn from these studies afford further support to the mode of bonding discussed on the basis of their 3D molecular modeling studies by considering different bond lengths, bond angles, and bond distance. The ligand and its metal complexes evaluated for their antimicrobial activity against *Staphylococcus aureus* (MTCC number 7443), *Bacillus subtilis* (MTCC number 9878), *Escherichia coli* (MTCC number 1698), *Aspergillus niger* (MTCC number 281), and *Aspergillus flavus* (MTCC number 277). The MIC of these compounds was found to be most active at 10 **μ**g/mL concentration in inhibiting the growth of the tested organisms. The DNA cleavage activity of all the complexes was studied by gel electrophoresis method.

## 1. Introduction

The antimicrobial resistances of the bacterial pathogens are of great anxiety on human health and well-being worldwide [1, 2]. In the treatment of animal and human patients with infectious disease antimicrobial resistance is considered to be a major problem. Recently a growing body of the literature has highlighted the dynamic nature of antimicrobial resistance in community pathogens [3]. Therefore to overcome the alarming problem of microbial resistance to antibiotics, the discovery of novel active compounds against new targets is a matter of urgency. Previously it was reported that quinazoline-4-(3H)-one derivatives have interesting antimicrobial activity against different species of gram-positive, gram-negative, and pathogenic fungi [4].

The extensive literature survey on heterocyclic compounds of Schiff bases and their metal complexes have been discussed by several researchers in coordination chemistry [5]; Schiff base ligands containing O, N-donor atoms have played a promising role in coordination chemistry owing to their remarkable complex forming ability with various metal ions. The formation of metal complexes with these Schiff base ligands is becoming increasingly important as biochemical, biomedical, analytical, and antimicrobial reagents [6].

Quinazoline-4(3H) is one of the most frequently encountered heterocycles in medicinal chemistry, which contains a pyrimidine nucleus in their structure, which often leads to pronounced biological and pharmaceutical activities especially antibacterial, antifungal, anti-inflammatory, anticancer, anticonvulsant, antioxidant, antitubercular, anti-HIV, and analgesic [7]. In addition, many substituted quinazoline derivatives have recently earned great interest in chemotherapy as antitumor drugs [8]. On the other hand, 2-hydroxy Schiff bases formed by condensation reactions of 2-hydroxy-1-naphthaldehyde with various amines have been extensively studied due to their wide applications particularly in pharmaceutical field [9]. In recent years people pay great interest in theoretical calculations of various Schiff base transition metal complexes in which interns played a fundamental role in the development of coordination compounds and also is helpful in designing and identification of new biological and chemotherapeutic agents [10].

In the absence of an X-ray crystal structure data the 3-dimensional structure of the molecules cannot be completely unambiguous. However, recent major advances in the computational chemistry tools provide an alternative, albeit approximate, approach for obtaining the three-dimensional structure of the compounds. Computational chemistry may be defined as the application of mathematical and theoretical principles to the solution of chemical problems [11]. Molecular modeling, a subset of computational chemistry, concentrate on predicting the behaviour of individual molecules within a chemical system. The most accurate molecular models use an initial or (first principles) electronic structure methods, based upon the principles of quantum mechanics, and generally vary computer intensive. Due to advances in computer storage capacity and processor performance, molecular modelling emerged as an interactive graphics program and has been a rapidly evolving and expanding field to the point that it is now possible to solve relevant problems in an acceptable amount of time that is. It allows rapid structure building, geometry optimization with minimum energy, and molecular display. It has the ability to handle transition metal and inner transition metal complexes [12, 13]. Theoretical calculations have paid a considerable attention to the characterization and inferences of geometrical optimization of the prepared complexes and it also enables the calculation of the actual bond lengths, angles, and total static energy of a molecule in terms of deviations from reference unstrained bond lengths, angles, and torsions plus nonbonded interactions [14, 15].

Hence in view of the analytical and biological importance of quinazoline-4(3H) and as a part of our continued work on the chemistry of quinazoline-4(3H)-one ring system [16], the present work deals with the synthesis and characterization of a new (E)-3-((2-hydroxynaphthalen-1-yl)methleneamino)-2-methylquinazoline-4(3H)-one HNMAMQ to be designed with various potential ligating sites by suitably orienting the groups such as N, O, >C=N, and OH for tridentate chelation with Cu(II), Co(II), Ni(II), Mn(II), Zn(II), and Cd(II) complexes in a way to produce interesting core structures.

In addition to that the available literature survey stimulated our interest to obtain structural information, optimized geometry of ligand HNMAMQ, and its complexes by computing the minimum satiric energy and the theoretical physical parameters, such as bond length and bond angles using MM2CS Chem 3D Ultra 11.0 version molecular modeling program; these models are necessary to obtain a constant, more precise picture of the biological active molecule at the atomic level and furthermore provide new insights that can be used to design novel therapeutic agents. The ligand HNMAMQ and its complexes were screened for their antimicrobial and DNA cleavage activities to check their biological potency.

## 2. Experimental

### 2.1. General Methods and Materials

All the reagents were commercially obtained from Hi-media (India). Elemental analysis (C, H, and N) was carried out using Flash EA 1112 series elemental analyzer. Infrared spectra of the ligand and its metal complexes in KBr pellets were recorded in the spectral range 4000–350 cm^−1^ using Perkin Elmer Spectrum-one FT-IR spectrometer. UV-visible spectra were recorded in the range 200–1100 nm on Elico SL-164 double beam UV-Vis spectrophotometer. The ^1^H NMR spectra were recorded on AMX-400 NMR spectrometer, using TMS as internal standard and DMSO as a solvent. ^13^C NMR spectra were recorded on Bruker ACF-125 MHz spectrometer. Mass spectra were recorded with a JEOL GC MATE II GC-MS mass spectrometer. Magnetic susceptibilities were measured on a Guoy balance at room temperature using Hg[Co(NCS)_4_] as calibrant. The molar conductance data were recorded on the ELICO-CM-82T conductivity bridge in DMF solution at concentration ~10^−3^ M and ESR spectra were recorded on Bruker Biospin. Interaction of metal complexes with DNA of *E. coli* was done in 0.01 M buffer (pH 7.2).

### 2.2. Chemistry

**Scheme 1:**
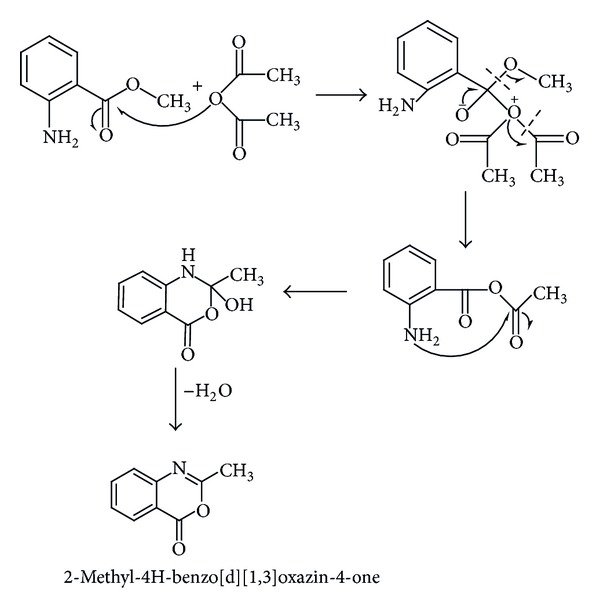


A simple and efficient synthetic strategy is employed for the preparation of new (E)-3-((2-hydroxynaphthalen-1-yl)methyleneamino)-2-methylquinazoline-4(3H)-one HNMAMQ Schiff base ligand as presented in Scheme 1, by involving the following three steps.

**Step 1 (preparation of 2-methyl-4H-benzo[d][1,3]oxazin-4-one)**

When a hot solution of methyl anthranilate reacts with acetic anhydride in 20 mL methanol results in the formation of a cyclic compound 2-methyl-4H-benzo[d][1,3]oxazin-4-one as a white silvery needle (see Scheme 1).

**Step 2 (preparation of 3-amino-2-methylquinazoline-4(3H)-one)**

**Scheme 2:**
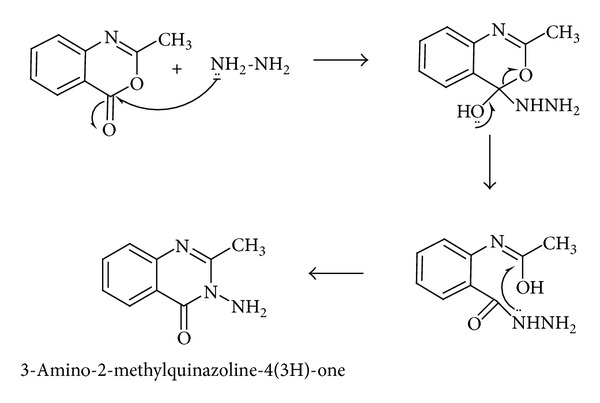


In Step 2 the reaction of 2-methyl-4H-benzo[d][1,3]oxazin-4-one with hydrazine in 20 mL hot methanol gives 3-amino-2-methylquinazoline-4(3H)-one [17] (see Scheme 2).

**Step 3 (preparation of (E)-3-((2-hydroxynaphthalen-1-yl)methyleneamino)-2-methylquinazoline-4(3H)-one HNMAMQ Schiff base ligand)**

**Scheme 3:**
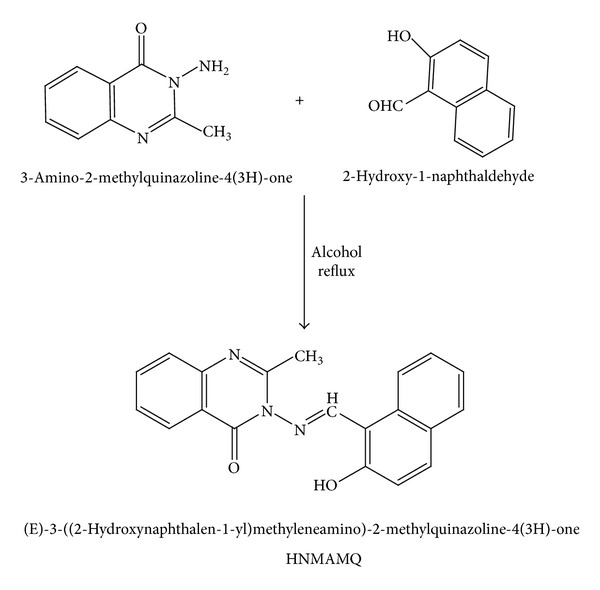
Synthetic route for the preparation of ligand HNMAMQ.

In Step 3 a simple condensation reaction between 3-amino-2-methyl quinazoline-4-one and 2-hydroxy-1-naphthaldehyde results in the formation of new Schiff base ligand with higher yield as presented in Scheme 3.

### 2.3. Synthesis of Ligand HNMAMQ

The synthetic route for the preparation of tridentate ligand HNMAMQ is as follows.

A 25 mL methanolic solution of both 3-amino-2-methylquinazoline-4-one (1.75 g, 0.01 mol) and 2-hydroxy-1-naphthaldehyde (1.72 g, 0.01 mol) was taken in 100 mL round bottom flask and heated to reflux for about 4-5 h on a water bath. The progress of the reaction was continuously checked with the aid of TLC. The coloured solid product obtained from the above reaction after evaporation of the solvent was filtered, washed with cold methanol, and recrystallized from hot methanol, to get ligand HNMAMQ, as presented in Scheme 3.

### 2.4. Synthesis of Metal(II) Complexes

A familiar method is used for the synthesis of metal complexes by using reaction between metal salts and ligand in a molar ratio (M : L = 1 : 1 and 1 : 2). An alcoholic solution of ligand HNMAMQ (35 mL, 0.01 mmol) and Cu(II), Ni(II) Co(II), Mn(II), Zn(II), and Cd(II) chlorides (10 mL, 0.01 mmol) were mixed vigorously and heated to reflux for 3 h. The volume of the reaction mixture was reduced to one-half by evaporation of solvent. Furthermore 0.5 g of sodium acetate was added to the reaction mixture to adjust the pH 7-8 followed by refluxing for about 2 h more. The solid thus obtained after cooling was filtered, washed thoroughly with hot methanol to apparent dryness, and finally dried in vacuum over fused CaCl_2_.

### 2.5. Study of Complex Formation in Solution

The complexes of the ligand HNMAMQ with metal ions were studied in DMF, in order to determine the M : L ratio in the complex following the molar ratio method. A series of solutions were prepared having a constant concentration (10^−3^ M) of the metal ions and (L). The M : L ratio was determined by the relationship between the absorption of light and the molar ratio of M : L. The results of complex formation in solution were presented in Table 1.

### 2.6. Biological Studies

#### 2.6.1. DNA Cleavage Studies

Preparation of culture media for the DNA cleavage studies of metal complexes and the isolation of DNA were carried out according to the literature procedure [18].

#### 2.6.2. Agarose Gel Electrophoresis

The DNA cleavage products were analyzed by agarose gel electrophoresis method [18]. The test solution of metal complexes was prepared by dissolving 10 mg of the compound in 10 mL of DMSO. The sample (25 *μ*g/mL) was added to the isolated DNA of *E. coli.* The samples were incubated for 2 h at 37°C, and then 20 *μ*L of DNA sample (mixed with bromophenol blue dye at a 1 : 1 ratio) was loaded carefully into the electrophoresis chamber wells along with a standard DNA marker in TAE buffer (4.84 g Tris base, pH 8.0; 0.5 M EDTA/1 L). Then a constant electricity (120 V) was applied for about 30 min. Finally, the gel was removed and stained with 10 *μ*g/mL of ethidium bromide for 10–15 min. The bands obtained were observed under the Vilber Lourmate gel documentation system and photographed to determine the extent of DNA cleavage. Then the results were compared with standard DNA marker.

#### 2.6.3. **In Vitro** Antimicrobial Activities

All the microorganisms used in the antimicrobial analysis were collected from microbial type culture collection (MTCC), Chandigarh, India. The antimicrobial activities of the ligand HNMAMQ and its complexes were tested against three bacteria and two fungi, such as (*Staphylococcus aureus* (MTCC number 7443), *Bacillus subtilis* (MTCC number 9878), *Escherichia coli* (MTCC number 1698), *Aspergillus niger* (MTCC number 281), and *Aspergillus flavus* (MTCC number 277)), respectively. To evaluate the antimicrobial activity we determined the minimum inhibitory concentration (MIC). The MIC was determined by taking different volumes (5, 10, 15, 20, 25, 30, 35, 40, 45, and 50 *μ*L) of same concentrations (1 mg/mL) of HNMAMQ and its complexes were added to Muller-Hinton broth containing the test organisms (maintained at 10^6^ CFU/mL) and incubated at 37°C for 24 h (bacteria) and 48 h (fungi). The MIC was determined by measuring the optical density at 625 nm.

## 3. Results and Discussion

In the present investigation, the new Schiff base ligand HNMAMQ and its Cu(II), Ni(II), Co(II), Mn(II), Zn(II), and Cd(II) complexes were synthesized by treating metal ions with ligand HNMAMQ. The evidence for the formation of tridentate ONO donor nature of ligand HNMAMQ was confirmed by using various spectral techniques (UV-visible, IR, NMR, and mass spectra). The analytical data and physical properties showed that the ligand HNMAMQ and its complexes were very stable and nonhygroscopic at room temperature. All the metal complexes were sparingly soluble in common organic solvents and show complete solubility in DMF and DMSO. The measured molar conductance values of the complexes were within the range of 9–20 Ohm^−1^ cm^2^ Mol^−1^ showing their nonelectrolytic nature [15]. Based on analytical and spectral studies, Cu(II), Ni(II) Co(II), and Mn(II) exhibits octahedral structures, while Zn(II) and Cd(II) complexes exhibits tetrahedral structures, where L stands for deprotonated legend. The metal content of the complexes was analyzed by decomposing with a mixture of HNO_3_ and HCl followed by H_2_SO_4_. The chloride was determined as AgCl by following a standard procedure [19]. The elemental analysis (C, H, and N) data of ligand HNMAMQ and its complexes were in good agreement with proposed molecular formulas.

### 3.1. Infrared Spectral Data of Ligand HNMAMQ and Its Complexes

The most prominent infrared bands for the ligand HNMAMQ and its complexes together with their assignments are listed in Table 2. The IR spectra of the ligand HNMAMQ exhibited a broadband in the region of 3425–3372 cm^−1^ due to the phenolic *ν*(OH), a characteristic high intensity band observed in the region of 1598–1592 cm^−1^ assigned to *ν*(C=N) and a high intensity strong band in the region of 1720–1715 cm^−1^ assigned to phenolic *ν*(C=O). Upon complexation with ligand HNMAMQ the IR spectra of metal complexes show the following changes. The absences of broad band due to phenolic *ν*(OH) ensure the involvement of phenolic oxygen in complex formation via deprotonation [20]. The band due to *ν*(C=N) group that was in the region of 1590–1578 cm^−1^ shows shift to the lower region which can be attributed to delocalisation of metal electron density (*t*_2g_) to the *π*-system of the ligand inferred the involvement of azomethine nitrogen [21]. Band appeared in the region of 1714–1704 cm^−1^ due to *ν*(C=O) indicates the participation of the phenolic oxygen in complexation with metal ion [22]. Furthermore, the metal complexes displayed *ν*(M–N) and *ν*(M–O) bands in the regions of 552–545 cm^−1^ and 460–447 cm^−1^ which give the evidence for the coordination through carboxylato oxygen and azomethine nitrogen of the ligand HNMAMQ with the metal ions [23, 24]. The diamagnetic Zn(II) and Cd(II) complexes exhibit a weak band due to *ν*(M–Cl) in the region of 357–352 cm^−1^ which was further confirmed by their quantitative analysis. Thus IR spectral data evidently confirms the coordination of ligand HNMAMQ with different metal ions.

### 3.2. ^****1****^H NMR and ^****13****^C NMR Spectral Studies

NMR spectra of ligand HNMAMQ and its Zn(II) and Cd(II) complexes were carried in DMSO-d6 in the presence of TMS as an internal standard. The ligand HNMAMQ displays a characteristic ^1^H NMR signal at *δ* 10.61 ppm (s, 1H, –OH) due to naphthalene phenolic –OH proton. The signal due to azomethine proton resonated at *δ* 8.35 ppm (s, 1H, –CH=N) and methyl protons at *δ* 2.41 ppm (s, 3H, CH_3_). The aromatic protons of the ligand HNMAMQ appeared as multiplets in the range of *δ* 6.82–8.54 ppm (m, 10H, Ar-H). The ligand HNMAMQ upon complexation with Zn(II) and Cd(II) ion shows a disappearance of signal due to phenolic-OH which indicates the involvement of phenolic oxygen [25]. The shift of the signal from *δ* 8.35 to *δ* 8.41–8.48 ppm due to azomethine proton indicates the coordination of azomethine nitrogen with metal ion [26]. The –CH_3_ protons appear at *δ* 2.55–2.60 ppm, while the aromatic protons appear as a multiplets in the range of *δ* 6.91–8.61 ppm.

The ^13^C NMR spectrum of ligand HNMAMQ exhibits signals at *δ* 163.32 ppm, *δ* 160.20 ppm, and *δ* 171.02 ppm due to azomethine carbon (HC=N), carbonyl carbon (C=O), and aromatic carbons (Ar-OH), respectively. However, the ^13^C NMR spectrum of Zn(II) and Cd(II) complexes shows a slight shift in their signals and resonates at *δ* 161.41–161.48 ppm, *δ* 173.41–173.50 ppm, and *δ* 154.82–155.12 ppm mainly due to azomethine carbon (HC=N) [27], carbonyl carbon (C=O), and aromatic carbons (Ar-OH), respectively.

Hence, from the above observations it was concluded that the total number of protons and carbons, calculated from the integration curves, and the values obtained from their C, H, and N analysis were in agreement with each other.

### 3.3. UV-Visible Spectral Studies and Magnetic Susceptibility Measurements

The Cu(II), Co(II), Ni(II), and Mn(II) complexes of ligand HNMAMQ were subjected to their UV-visible spectral studies at room temperature in the range of 200–1100 nm by using DMSO as a solvent. The Cu(II) complex displays a asymmetric broadband at 12530–17020 cm^−1^. The ^2^E_g_ and ^2^T_2g_ states of the octahedral Cu(II) complex split under the influence of tetrahedral distortion and cause three transitions: ^2^B_1g_ → ^2^A_1g_  (d_*x*^2^−*y*^2^_ → d_*z*^2^_)(*ν*_1_), ^2^B_1g_ → ^2^B_2g_  (d_*x*^2^−*y*^2^_ → d_*zy*_)(*ν*_2_), and ^2^B_1g_ → ^2^E_g_  (d_*x*^2^−*y*^2^_ → d_*zy*_, d_*yz*_)(*ν*_3_) having equal energy and show only one broad absorption band due to dynamic Jahn-Teller distortion in which interns confirm the distorted octahedral geometry around the Cu(II) ion [28]. The Co(II) complex shows two bands at 18337 and 20532 cm^−1^ due to ^4^T_1g_(F) → ^4^A_2g_(F)(*ν*_2_) and ^4^T_1g_(F) → ^4^T_2g_(F)(*ν*_3_) transitions, showing the octahedral stereochemistry around Co(II) metal ion [29]. However, the lower energy *ν*_1_ band could be calculated by using Underhill and Billing equations [30]. The Ni(II) complex that displays three bands at 8213, 15850, and 20430 cm^−1^ due to ^3^A_2g_(F) → ^3^T_2g_(F)(*ν*_1_), ^3^A_2g_(F) → ^3^T_1g_(F)(*ν*_2_), and ^3^A_2g_(F) → ^3^T_1g_(P)(*ν*_3_) transitions reveals the octahedral geometry around the Ni(II) ion [31]. The Mn(II) complex that exhibits bands at 10120, 19010, and 21820 cm^−1^ assigned due to ^6^A_1g_ → ^4^T_1g_(*ν*_1_), ^6^A_1g_ → ^4^T_2g_(*ν*_2_), and ^6^A_1g_ → ^4^E_g_(*ν*_3_) transition indicates the coordinated octahedral geometry [32].

Table 1 shows the measured magnetic moment values of Cu(II), Co(II), Ni(II), and Mn(II) complexes in the range of 1.80–1.85 BM, 3.34–3.62 BM, 4.39–4.60 BM, and 5.62–5.90 BM, respectively, and indicates their octahedral geometries [33]. Furthermore, the values of electronic parameters such as the Racah interelectronic repulsion parameter (*B*′), covalency factor (*β*), ligand field splitting energy (10 Dq), and ligand field stabilization energy (LFSE) [34] were summarized in Table 3. In addition, from the values of *β*, the Covalence factors (*b* ^1/2^), Sinha parameter (*δ*%), that is, Metal-ligand covalency percent, and the covalency angular overlap parameter (*η*) have been calculated using the following three expressions [35]:

(1) $$\begin{array}{c} b^{1/2}=\frac{1}{2}\left[{\left(1-\beta \right)}^{1/2}\right],\;\;\delta \left(\%\right)=\left[\frac{\left(1-\beta \right)}{\beta }\right]\times 100, \end{array}$$

(2) $$\begin{array}{c} \eta \;=\;\frac{\left[\left({1-\beta }^{1/2}\right)\right]}{\beta^{1/2}}. \end{array}$$

The positive values for (1 − *β*), *b* ^1/2^, *δ*%, and *η* indicate strong covalent bonding between ligand HNMAMQ and its complexes.

### 3.4. Mass Spectral Studies

The mass spectrum of ligand HNMAMQ shows the formation of a molecular ion peak at *m/z* = 329 [M]^+^ equivalent to its total molecular weight. The mass spectrum of Zn(II) complex shows the molecular ion peaks at *m/z* = 429 [M]^+^ and 431 [M+2]^+^ which were in good agreement with the expected values, supporting the composition of the complex.

### 3.5. Electron Spin Resonance (ESR) Spectra of Cu(II) Complex

The ESR spectrum pattern of Cu(II) complex gave the following data g_||_ = 2.23 and g_⊥_ = 2.05, g_av_ = 2.11 and g_iso_ = 2.17. The observed g_||_ value was less than 2.3, in accordance with the covalent character of the metal-ligand bond. The ESR spectra showed asymmetric bands with g_||_ > g_⊥_ > 2.0023, suggest the presence of unpaired electron in d_*x*^2^−*y*^2^_ orbit, and indicate the octahedral geometry around Cu(II) ion [36]. The G parameter was calculated by using a formula G = (g_||_ − 2.0023)/(g_⊥_ − 2.0023) which measures the negligible exchange interaction between the metal centres in polycrystalline solid. In the present study the Cu(II) complex which shows G = 4.77 which was greater than 4 indicates the negligible exchange interaction in solid complex as suggested by Hathaway and Billing [37].

### 3.6. Molecular Modeling Studies of Ligand HNMAMQ and Its Complexes

An attempt to gain better insight on the molecular structure of the ligand and its complexes, geometric optimization, conformational analysis, and 3D molecular modeling of the proposed structure of the ligand HNMAMQ and its complexes was performed using MM2CS Chem 3D Ultra 11.0 version software and using pm3 programme.

The correct stereochemistry was assured through the exploitation and modification of the molecular coordinates to attain reasonable low energy molecular geometries. The potential energy of the molecule was considered as the sum of the following stipulations:

(3) $$\begin{array}{c} E=E_{\text{str}}+E_{\text{ang}}+E_{\text{tor}}+E_{\text{vdw}}+E_{\text{oop}}+E_{\text{ele}}, \end{array}$$

where *E* represents the energy values corresponding to the given type of interaction (kcal/mol). The subscripts str, ang, tor, vdw, oop, and ele signify bond stretching, angle bonding, torsion deformation, van der Waals interactions, out of plane bending, and electronic interaction, respectively. The analytical and spectral studies represent the hexacoordination [Cu(II), Ni(II), Co(II), and Mn(II)] and tetracoordination [Zn(II) and Cd(II)] of the complexes which were further confirmed by their molecular modeling studies.

**Figure 1:**
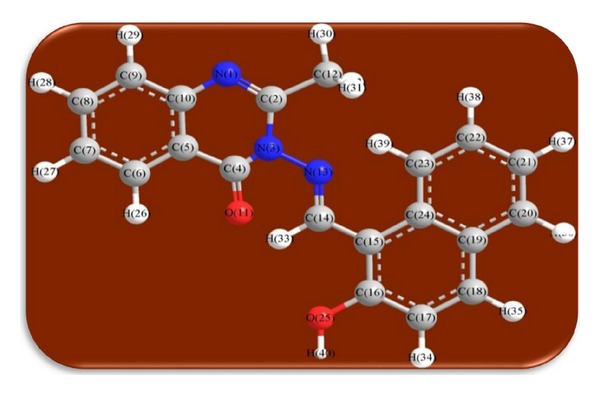
Molecular modeling structure of ligand HNMAMQ.

**Figure 2:**
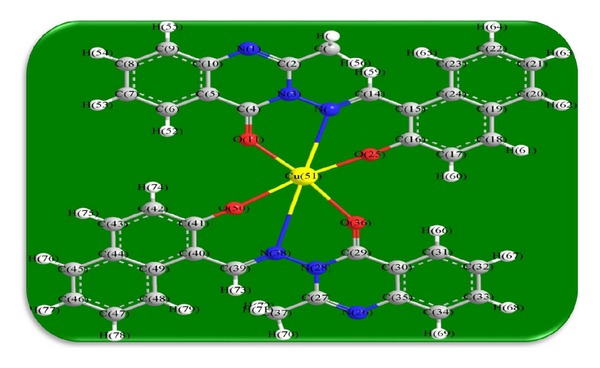
Molecular modeling structure of Cu(II) complex.

**Figure 3:**
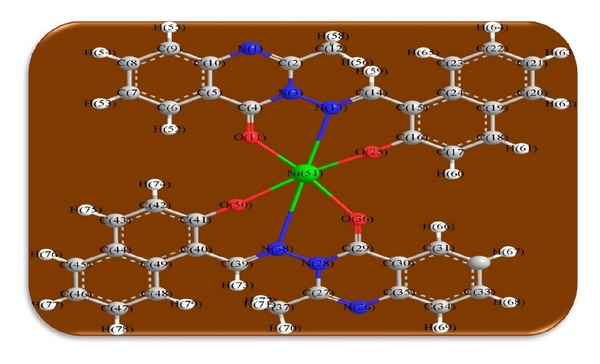
Molecular modeling structure of Ni(II) complex.

**Figure 4:**
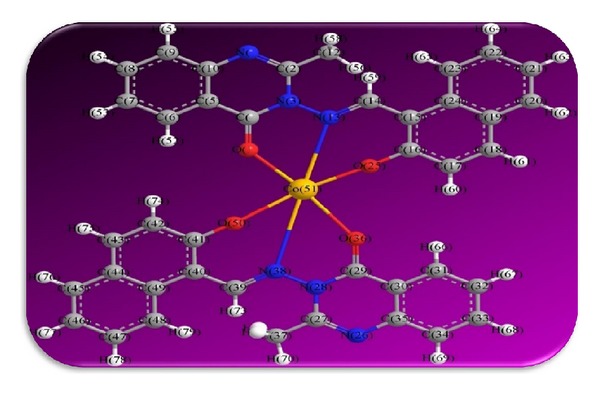
Molecular modeling structure of Co(II) complex.

**Figure 5:**
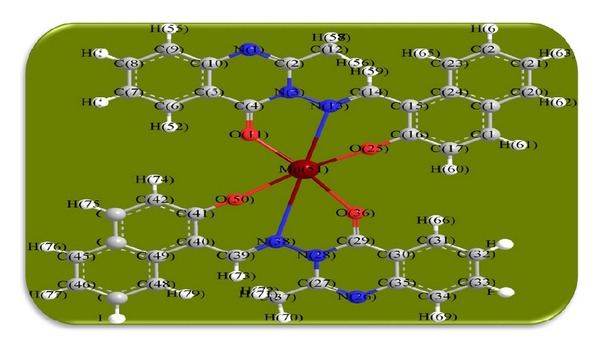
Molecular modeling structure of Mn(II) complex.

**Figure 6:**
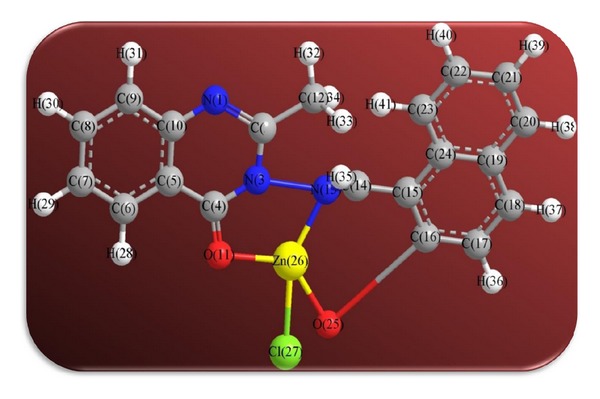
Molecular modeling structure of Zn(II) complex.

**Figure 7:**
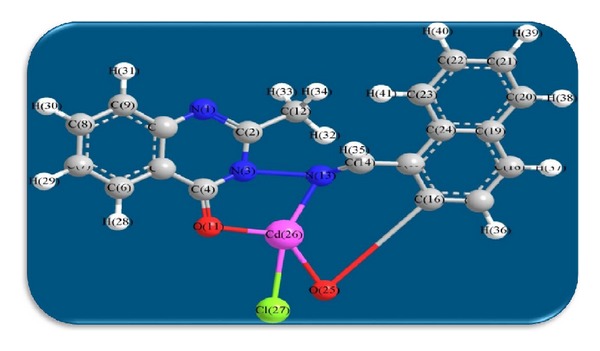
Molecular modeling structure of Cd(II) complex.

The 3D optimized geometrical structures of ligand HNMAMQ and its Cu(II), Ni(II), Co(II), Mn(II), Cd(II), and Zn(II) complexes were presented in Figures 1, 2, 3, 4, 5, 6, and 7. The minimum steric energy which was repeated several times to find out the global minimum obtained to be 23.474, 122.793, 143.906, 185.32, 128.35, 132.639, and 140.429 kcal/mole, respectively. The obtained bond lengths of the ligand HNMAMQ were in-between N(13)–C(14) 1.2150 Å, N(3)-N(13) 1.3552 Å, C(4)–O(11) 1.2610 Å, and C(16)–O(25) 1.3550 Å. From the analysis of the data in Tables S1–S3 The selected bond lengths and bond angles of optimized ligand HNMAMQ and its complexes were mentioned in Supplementary file (see Supplementary Material available online at http://dx.doi.org/10.1155/2014/817365) calculated for the bond lengths and bond angles, one can conclude the following annotations.

1. The N(3)–N(13) and C(4)–N(13) bond lengths become somewhat longer owing/to the coordination with ligand HNMAMQ which takes place via N atoms of the azomethine (>C=N) group that was formed on enolization of (CO) group in all complexes [38]. Also, the C(4)–O(11) bond distances become longer due to the formation of the M–O bond which makes the C–O bond weaker [39].
2. The Zn–N and Cd–N bond lengths obtained in the present calculations were closer to the earlier reported values to form single crystal X-ray studies to lie between 2.008 and 2.065 Å [40].
3. In complexes, the C(16)–O(25) bond distance becomes longer due to the deprotonation at N(13) which leads to a higher single bond character for C(16)–O(25) result in the formation of the M–O bond which makes the C–O bond weaker. In addition to that, the C(4)–N(3) and N(3)–N(13) bond distances were elongated. This refers to the formation of the M–N bond which makes the C–N bond weaker and forming a double bond character [41].
4. The bond angles in Cu(II), Ni(II), Co(II), and Mn(II) complexes were quite near to an octahedral geometry predicting d^2^sp^3^ hybridization, while Zn(II) and Cd(II) complexes afforded a tetrahedral geometry predicting sp^3^ hybridization.

Hence from the above, it was inferred that, when the ligand HNMAMQ containing donor atoms was coordinated with the metal ions by donating a lone pair of electrons results in decrease of electron density on the coordinating atoms, at the same time increase in the bond lengths and bond distance; that is, all the active groups participating in coordination have bonds longer than those already exist in the ligand HNMAMQ (like C=N, C=O, and O–H). Furthermore in most of the cases, the actual bond angles and lengths are close to the optimal values (most favourable), thereby supporting the proposed tetra- and hexacoordinated geometry around the metal ion of these compounds [42].

### 3.7. Theoretical Structural Models: Surface Area and Charge Density of Ligand HNMAMQ and Its Cu(II) Complex

The solvent-accessible surface (SAS) area is referred as the surface area of a biomolecule that is accessible to a solvent on the other hand the solvent-excluded surface (SES) also known as the molecular surface or Connolly surface, which is imagined as a cavity in bulk solvent effectively the inverse of the solvent-accessible surface, generally both are calculated using the “rolling ball” algorithm, but the difference is that in a SAS, the surface is drawn from the centre point of the probe radius, while in an SES, the surface is drawn from the touching point of the probe radius [43, 44].

Charge (electron) density is the measure of the probability of an electron being present at a specific location and it is usually found around the atom and its bonds. However, in delocalized or conjugated systems, it covers an entire region; that is, in benzene they are found above and below the planar ring. Molecular modeling software often provides graphical images of electron charge density by representing the presence of high or low electron density in a particular position of molecules because graphically the electron density surface serves as a canvas upon which other electronic properties can be displayed as follows.

1. The electrostatic potential map (the property of electrostatic potential mapped upon the electron density) provides an indicator for charge distribution in a molecule.
2. The local ionization potential map (the property of local ionization potential mapped upon the electron density) provides an indicator of electrophilicity.
3. The LUMO map (lowest unoccupied molecular orbital mapped upon the electron density) can provide an indicator for nucleophilicity [45].

**Figure 8:**
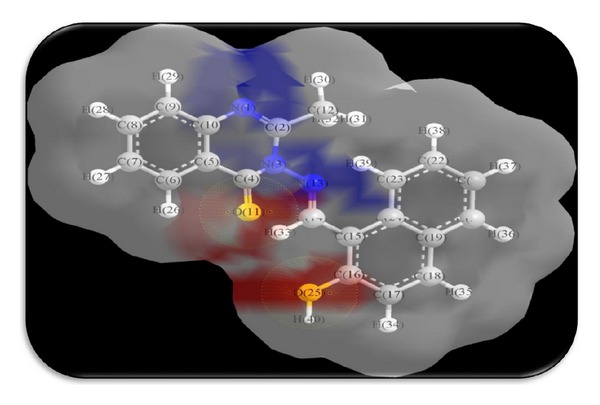
Solvent accessible surface of ligand HNMAMQ.

**Figure 9:**
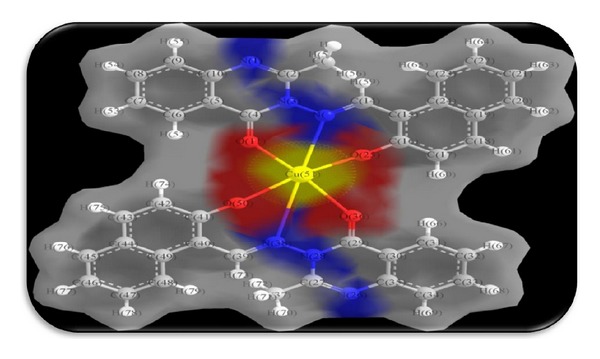
Solvent accessible surface of Cu(II) complex.

**Figure 10:**
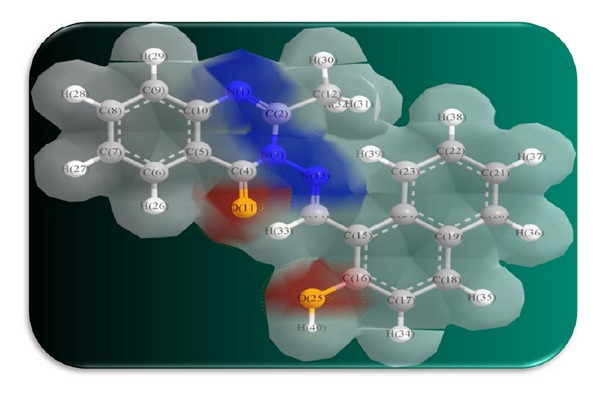
Connolly molecular surface of ligand HNMAMQ.

**Figure 11:**
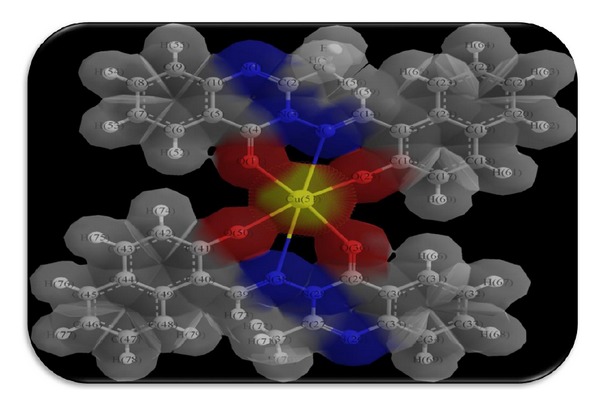
Connolly molecular surface of Cu(II) complex.

**Figure 12:**
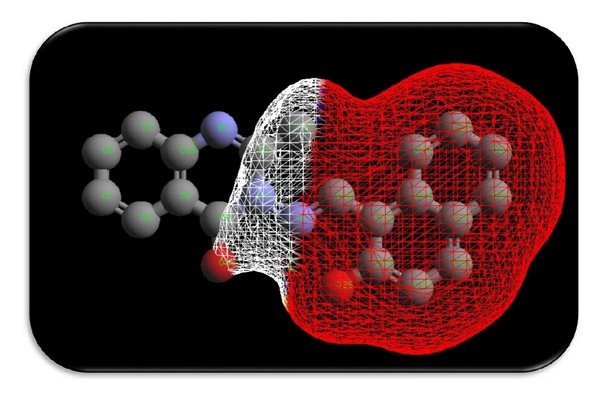
Total charge density of ligand HNMAMQ.

**Figure 13:**
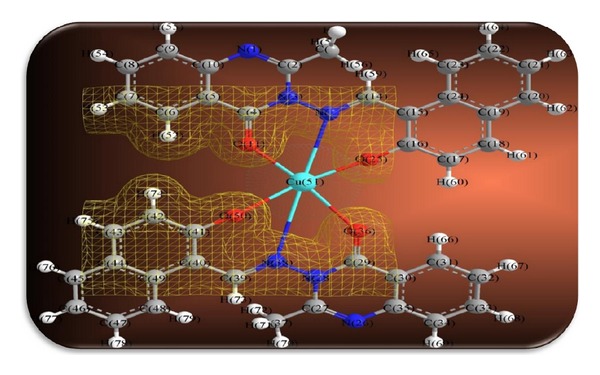
Total charge density of Cu(II) complex.

To know the interaction of ligand HNMAMQ and its complexes, we thought of interest to draw the theoretical models of solvent-accessible surface (SAS), solvent-excluded surface (SES), and total charge density especially for the ligand HNMAMQ and its Cu(II) complex as shown in Figures 8, 9, 10, 11, 12, and 13. Further calculating their theoretical structural analysis results is our next goal.

### 3.8. Biological Results

#### 3.8.1. DNA Cleavage Studies

All the metal complexes of ligand HNMAMQ were studied for their DNA cleavage activity by gel electrophoresis. From the gel picture study it was clearly observed that all the metal complexes show complete DNA cleavage. The variation in the bands of complexes (lanes I–VI) was observed as compared with control DNA of *E. coli.* In general, metal complexes upon binding to DNA get stabilized through a series of weak interactions such as the stacking interactions of aromatic heterocyclic groups between the base pairs, hydrogen bonding, and van der Waals interactions of functional groups that bound along the groove of the DNA helix [46]. The DNA cleavage activity was significantly increased by the inclusion of metal ion in the Schiff base. From these results it was accomplished that the control DNA alone does not show any apparent cleavage, whereas its complexes show superior activity and also indicate the vital role of metal ions in these isolated DNA cleavage reactions. However, the nature of the reactive intermediates involved in the DNA cleavage by the complexes is not clear. As the compound was observed to cleave the DNA, it can be concluded that the compound inhibits the growth of the pathogenic organism by cleaving the genome [47].

#### 3.8.2. **In Vitro** Antimicrobial Activities

The *in vitro* antibacterial results reveal that the ligand HNMAMQ and its complexes were found to be potentially active against *E. coli* (MTCC number 1698) and moderately active against *S. aureus* (MTCC number 7443) and *B. subtilis* (MTCC number 9878). This may be due to inhibitory activity of ligand HNMAMQ related to the cell wall structure of the bacteria. In general, the membrane of gram-negative bacteria was surrounded by an outer membrane containing lipopolysaccharides. The ligand HNMAMQ appears to be able to combine with the lipophilic layer to increase the membrane permeability of the gram-negative bacteria. Therefore the lipophilicity plays a significant role in causing the death of the gram-negative bacteria [48]. Moreover, the synergistic effect of ligand (HAMAMQ) with metal complexes enhances the antimicrobial activity; this may be explained by chelation theory. On chelation the positive charge of metal is partially shared with the donor atoms present in the ligands; there may be *π*-electron delocalization over the whole chelating. This increases the lipophilic character of the metal chelate and favours its penetration through the lipid layer of the bacterial membranes. Chelation is not only the criterion for antibacterial activity; it is expected to be a function of steric, electronic, and pharmacokinetic factors along with mechanistic pathway. Other factors such as solubility, conductivity, dipole moment, size of metal ions, stability constants of the complexes, and their magnetic moments are also reported to affect the microbial activity of the complexes [49]. This enhancement in the activity is also rationalized due to presence of azomethine (C=N) group; this imports in elucidating the mechanism of transamination and resamination reactions in biological system. In addition to this the formation of hydrogen bonds through the azomethine group with the active centres of various cellular constituents, resulting in interference with normal cellular processes [50]. Furthermore, the ligands with nitrogen and oxygen donor systems might inhibit enzyme production, since the enzymes which require these groups for their activity appear to be especially more susceptible to deactivation by the metal ions upon chelation [51].

In case of antifungal activity, the ligand (HAMAMQ) exhibits good activity against *A. flavus* (MTCC number 277) than *A. niger* (MTCC number 281). Among all the metal complexes Cu(II) shows promising activity than the other metal complexes.

Table 4 represents the MIC results of the antimicrobial activities of ligand (HAMAMQ) and its complexes, this shows that Cu(II) complex shows superior activity, Ni(II), Mn(II), Cd(II), and Co(II) complexes exhibit good activity, and Zn(II) complex exhibits moderate antimicrobial activity. The enhanced activity of Cu(II) complex can be explained on the basis of their particle size and also may be attributed to its higher stability constants [52].

Thus from the above data, it is of interest to note that some of the mononuclear complexes shows promising activity which interns reflecting the biological efficiency of these complexes, as a useful novel pharmaceutical agents.

## 4. Conclusion

**Figure 14:**
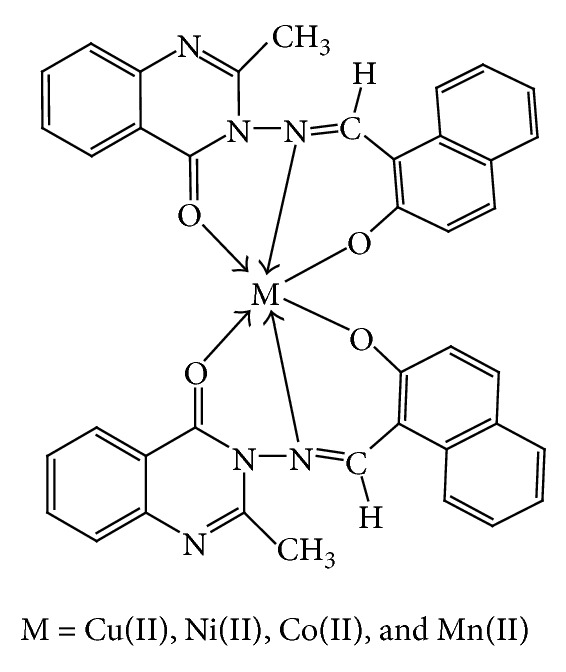
Proposed structures of Cu(II), Ni(II), Co(II), and Mn(II) complexes.

**Figure 15:**
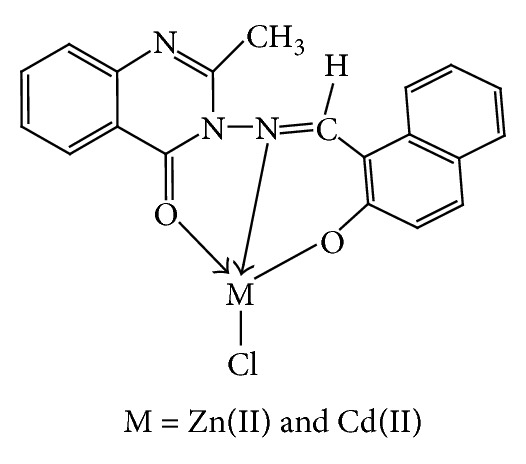
Proposed structures of Zn(II) and Cd(II) complexes.

In the present study we described the tridentate behaviour of ligand HNMAMQ which forms an octahedral geometry with Cu(II), Ni(II), Co(II), and Mn(II) complexes, whereas tetrahedral geometry with Zn(II) and Cd(II) complexes through the involvement of azomethine nitrogen, carboxylato oxygen, and phenolic oxygen donor atoms is based on their analytical, spectral, magnetic, and molecular modeling studies (Figures 14 and 15). The nonelectrolytic nature of the complexes was confirmed on the basis of their molar conductance values. The DNA cleavage studies revealed that all metal complexes showed good efficiency towards DNA cleavage. In addition to that, in case of antimicrobial activty, the Cu(II), Ni(II), Mn(II), and Cd(II) complexes show remarkable activity than the ligand HNMAMQ. Therefore, the antimicrobial results are extended to develop new metallodrug as an alternative to existing antibiotics used to treat the drug resistant microbial pathogens. The molecular modeling studies reflect the theoretical important application of these newly synthesized compounds in coordination chemistry that is, in determining the correct bond length, bond angle as well as geometry, and so forth, and also open up the several opportunities to explore the biological potency of these compounds. Hence from all these extensive observations, it was concluded that the ligand HNMAMQ and its complexes give the remarkable, versatile, and valuable information of coordination compounds in analytical and medicinal field and also they may be used as potent biological agents with reduced toxicity and higher efficiency.

## Supplementary Material

The selected bond lengths and bond angles of optimized ligand HNMAMQ and its complexes mentioned in Supplementary file.

## Figures and Tables

**Table 1:** Elemental analysis, magnetic susceptibility, and molar conductance data of ligand HNMAMQ and its complexes.

Compounds	Mol. wt. (g/mol)	Color	Mp. (°C)	Elemental analysis found and calculated %					M : L	*μ*_eff_ (BM)	Λ_m_*
				C	H	N	M	Cl			
C_20_H_15_N_3_O_2_ (HNMAMQ)	329.35	Brown	270	72.24 (72.94)	4.12 (4.59)	11.97 (12.76)	—	—	—	—	—
[Cu(C_20_H_14_N_3_O_2_)_2_]	720.23	Green	292	65.96 (66.70)	3.38 (3.92)	11.12 (11.67)	8.35 (8.82)	—	1 : 2	1.82	20.10
[Ni(C_20_H_14_N_3_O_2_)_2_]	715.38	Light green	285	66.78 (67.16)	3.11 (3.92)	11.28 (11.75)	7.86 (8.20)	—	1 : 2	3.42	12.54
[Co(C_20_H_14_N_3_O_2_)_2_]	715.62	Pink	283	66.70 (67.13)	3.37 (3.94)	11.14 (11.74)	7.75 (8.24)	—	1 : 2	4.47	10.75
[Mn(C_20_H_14_N_3_O_2_)_2_]	711.63	Grey	287	67.02 (67.51)	3.45 (3.97)	11.27 (11.81)	7.18 (7.72)	—	1 : 2	5.81	13.32
[Zn(C_20_H_14_N_3_O_2_)Cl]	429.21	White	285	55.67 (55.97)	2.96 (3.29)	9.20 (9.79)	14.98 (15.24)	7.92 (8.26)	1 : 1	Diam	15.52
[Cd(C_20_H_14_N_3_O_2_)Cl]	476.21	Pink	290	50.27 (50.44)	2.76 (2.96)	8.60 (8.82)	23.52 (23.61)	7.22 (7.44)	1 : 1	Diam	9.20

*Molar conductance values in Ohm^−1^ cm^2^ mol^−1^.

**Table 2:** The infrared frequencies (in cm^−1^) of ligand HNMAMQ and its complexes.

Compounds	*ν*_OH/H_2_O_	*ν*_C=O_	*ν*_C=N_	*ν*_C–O_	*ν*_M–O_	*ν*_M–N_	*ν*_M–Cl_
C_20_H_15_N_3_O_2_ (HNMAMQ)	3425	1720	1598	1287	—	—	—
[Cu(C_20_H_14_N_3_O_2_)_2_]	—	1705	1580	1312	550	460	—
[Ni(C_20_H_14_N_3_O_2_)_2_]	—	1714	1582	1310	545	447	—
[Co(C_20_H_14_N_3_O_2_)_2_]	—	1695	1590	1317	552	455	—
[Mn(C_20_H_14_N_3_O_2_)_2_]	—	1698	1578	1304	547	452	—
[Zn(C_20_H_14_N_3_O_2_)Cl]	—	1704	1581	1311	548	468	353
[Cd(C_20_H_14_N_3_O_2_)Cl]	—	1700	1584	1317	553	461	355

**Table 3:** Ligand field, Sinha, metal-ligand covalency percent, and covalency angular overlap parameters of Cu(II), Ni(II), Co(II), and Mn(II) complexes.

Complexes	Dq	*B*′	*β*	*β*%	*ν*_2_/*ν*_1_	(1−*β*)	*b* ^1/2^	*δ*%	*η*	LFSC (Kcal)
[Cu(C_20_H_14_N_3_O_2_)_2_]	1514	—	—	—	—	—	—	—	—	25.25
[Ni(C_20_H_14_N_3_O_2_)_2_]	930	891	0.91	8.2	1.92	0.09	0.15	9.89	0.31	15.94
[Co(C_20_H_14_N_3_O_2_)_2_]	941	819	0.84	15.6	1.93	0.16	0.20	19.04	0.43	16.13
[Mn(C_20_H_14_N_3_O_2_)_2_]	912	944	0.97	2.7	1.87	0.03	0.08	3.09	0.17	15.63

**Table 4:** MIC (*μ*g/mL) of ligand HNMAMQ and its complexes.

Compounds	*S. aureus* (MTCC number 7443)	*B. subtilis* (MTCC number 9878)	*E. coli* (MTCC number 1698)	*A. flavus* (MTCC number 281)	*A. niger* (MTCC number 277)
C_20_H_15_N_3_O_2_ (HNMAMQ)	45	40	25	50	50
[Cu(C_20_H_14_N_3_O_2_)_2_]	15	20	10	10	15
[Ni(C_20_H_14_N_3_O_2_)_2_]	25	20	15	15	15
[Co(C_20_H_14_N_3_O_2_)_2_]	25	20	20	15	20
[Mn(C_20_H_14_N_3_O_2_)_2_]	15	15	15	20	15
[Zn(C_20_H_14_N_3_O_2_)Cl]	35	30	25	45	45
[Cd(C_20_H_14_N_3_O_2_)Cl]	15	15	15	20	15
*Gentamicine *	10	11	10	—	—
*Fluconazole *	—	—	—	10	10
