# Supplementary material for: Spectral Characterization and 3D Molecular Modeling Studies of Metal Complexes Involving the O, N-Donor Environment of Quinazoline-4(3H)-one Schiff Base and Their Biological Studies
Source: ScientificWorldJournal. 2014 Feb 11;2014:817365. doi: 10.1155/2014/817365 (PMC3942396; doi:10.1155/2014/817365)
Supplement: Supplementary file 1 — The selected bond lengths and bond angles of optimized ligand HNMAMQ and its complexes mentioned in Supplementary file. [file 817365.f1.docx]

|  |  | Bond lengths | |  | Bond angles | |
| --- | --- | --- | --- | --- | --- | --- |
| No | Bonded atoms | Actual | Optimized | Bonded atoms | Actual | Optimized |
| 1 | C(12)-H(32) | 1.1135 | 1.1130 | O(25)-H(40) | 0.9707 | 0.9720 |
| 2 | C(12)-H(31) | 1.1133 | 1.1130 | N(3)-N(13)-C(14) | 119.1873 | 1.1000 |
| 3 | C(9)-H(29) | 1.1042 | 1.1000 | N(13)-C(14) | 1.5898 | 1.2600 |
| 4 | C(16)-O(25) | 1.3504 | 1.3550 | C(14)-C(15) | 1.5070 | 1.5030 |
| 5 | N(13)-C(14) | 1.2163 | 1.2150 | C(16)-O(25) | 1.3582 | 1.3550 |
| 6 | N(3)-N(13) | 1.3550 |  | N(13)-C(14) | 1.2642 | 1.2608 |
| 7 | C(4)-O(11) | 1.2645 | 1.2610 | N(3)-N(13) | 1.3551 |  |
| 8 | C(19)-C(24) | 1.4559 | 1.4200 | C(4)-O(11) | 1.2047 | 1.2080 |
| 9 | C(15)-C(24) | 1.5046 | 1.4200 | N(3)-C(4) | 1.3726 | 1.3690 |
| 10 | C(20)-C(21) | 1.5061 | 1.4200 | O(25)-C(16)-C(15) | 120.4214 | 124.3000 |
| 11 | C(19)-C(20) | 1.5100 | 1.4200 | C(10)-C(9)-C(8) | 117.6096 |  |
| 12 | C(4)-C(5) | 1.5116 | 1.5170 | H(26)-C(6)-C(7) | 118.9917 | 120.0000 |
| 13 | N(3)-C(4) | 1.3731 | 1.3690 | N(13)-N(3)-C(4) | 117.8898 |  |
| 14 | C(2)-N(3) | 1.3521 | 1.3450 | O(11)-C(4)-N(3) | 122.7259 | 122.6000 |
| 15 | N(1)-C(2) | 1.2712 | 1.2600 | C(12)-C(2)-N(1) | 116.1883 | 115.1000 |

**TABLE S1:** Some selected bond lengths and bond angles of optimized ligand (HNMAMQ).

**TABLE S:** Some selected bond lengths of optimized Cu(II) and Ni(II) complexes.

| Bond lengths (A°) | | | | | | |
| --- | --- | --- | --- | --- | --- | --- |
| No | Bonded atoms | [Cu(C_20_H_14_N_3_O_2_)_2_] | | Bonded atoms | [Ni(C_20_H_14_N_3_O_2_)_2_] | |
|  |  | Actual | Optimized |  | Actual | Optimized |
| 1 | C(48)-H(79) | 1.1000 | 1.1000 | C(46)-H(77) | 1.1007 | 1.1000 |
| 2 | N(38)-C(39) | 1.2600 | 1.2602 | C(48)-H(79) | 1.0994 | 1.1000 |
| 3 | C(27)-C(37) | 1.4970 | 1.4970 | C(12)-H(56) | 1.1117 | 1.1130 |
| 4 | C(16)-O(25) | 1.3629 | 1.3590 | C(9)-H(55) | 1.1045 | 1.1000 |
| 5 | N(13)-C(14) | 1.2595 | 1.2608 | C(16)-O(25) | 1.3774 | 1.3550 |
| 6 | N(3)-N(13) | 1.9631 | 1.9260 | N(13)-C(14) | 2.1982 | 1.2608 |
| 7 | C(4)-O(11) | 1.2956 | 1.2886 | N(3)-N(13) | 1.7102 | 1.4260 |
| 8 | C(22)-H(64) | 1.1000 | 1.1000 | C(4)-O(11) | 1.2974 | 1.2087 |
| 9 | C(6)-H(52) | 1.1000 | 1.1000 | C(29)-O(36) | 1.2193 | 1.2080 |
| 10 | O(36)-Cu(51) | 1.8100 |  | O(36)-Ni(51) | 1.7956 |  |
| 11 | N(38)-Cu(51) | 1.3030 | 1.3030 | N(38)-Ni(51) | 1.8493 |  |
| 12 | N(13)-Cu(51) | 1.3030 | 1.3030 | N(13)-Ni(51) | 1.8548 |  |
| 13 | O(11)-Cu(51) | 1.8100 |  | O(11)-Ni(51) | 1.7942 |  |
| 14 | O(25)-Cu(51) | 1.8100 |  | O(25)-Ni(51) | 1.7997 |  |
| 15 | O(50)-Cu(51) | 1.8100 |  | O(50)-Ni(51) | 1.7954 |  |

**TABLE S3:** Some selected bond length of optimized Co(II) and Mn(II) complexes.

| Bond lengths (A°) | | | | | | |
| --- | --- | --- | --- | --- | --- | --- |
| No | Bonded atoms | [Co(C_20_H_14_N_3_O_2_)_2_] | | Bonded atoms | [Mn(C_20_H_14_N_3_O_2_)_2_] | |
|  |  | Actual | Optimized |  | Actual | Optimized |
| 1 | C(22)-H(64) | 1.1000 | 1.1000 | C(42)-H(74) | 1.1027 | 1.1000 |
| 2 | C(21)-H(63) | 1.1000 | 1.1000 | C(39)-H(73) | 1.1000 | 1.1000 |
| 3 | N(38)-C(39) | 1.2600 | 1.2600 | C(37)-H(72) | 1.1161 | 1.1130 |
| 4 | C(16)-O(25) | 1.3731 | 1.3550 | C(6)-H(52) | 1.1028 | 1.1000 |
| 5 | N(13)-C(14) | 2.0038 | 1.2600 | C(16)-O(25) | 1.3721 | 1.3550 |
| 6 | N(3)-N(13) | 1.4510 | 1.4260 | N(13)-C(14) | 1.3328 | 1.2083 |
| 7 | C(4)-O(11) | 1.3199 | 1.2080 | N(3)-N(13) | 2.2719 | 1.4260 |
| 8 | C(41)-O(50) | 1.3551 | 1.3550 | C(4)-O(11) | 1.2974 | 1.2087 |
| 9 | C(45)-C(46) | 1.4200 | 1.4200 | C(44)-C(49) | 1.4210 | 1.4200 |
| 10 | O(36)-Co(51) | 0.9492 | 0.6000 | O(36)-Mn(51) | 1.8192 |  |
| 11 | N(38)-Co(51) | 1.8969 |  | N(38)-Mn(51) | 1.8769 |  |
| 12 | N(13)-Co(51) | 1.8980 |  | N(13)-Mn(51) | 1.8690 |  |
| 13 | O(11)-Co(51) | 0.9079 | 0.6000 | O(11)-Mn(51) | 1.8330 |  |
| 14 | O(25)-Co(51) | 1.8378 |  | O(25)-Mn(51) | 1.8222 |  |
| 15 | O(50)-Co(51) | 1.8402 |  | O(50)-Mn(51) | 1.8270 |  |

**TABLE S4:** Some selected bond length of optimized Zn(II) and Cd(II) complexes.

| Bond lengths (A°) | | | | | | |
| --- | --- | --- | --- | --- | --- | --- |
| No | Bonded atoms | [Zn(C_20_H_14_N_3_O_2_)Cl] | | Bonded atoms | [Cd(C_20_H_14_N_3_O_2_)Cl] | |
|  |  | Actual | Optimized |  | Actual | Optimized |
| 1 | C(46)-H(77) | 1.1000 | 1.1000 | C(7)-H(29) | 1.1000 | 1.1000 |
| 2 | C(48)-H(79) | 1.1000 | 1.1000 | C(6)-H(28) | 1.1000 | 1.1000 |
| 3 | C(21)-C(22) | 1.3372 | 1.3360 | N(38)-C(39) | 1.2600 | 1.2600 |
| 4 | C(20)-C(21) | 1.3370 | 1.3372 | C(16)-O(25) | 1.3731 | 1.3550 |
| 5 | C(16)-O(25) | 1.3703 | 1.3650 | N(13)-C(14) | 2.0038 | 1.2600 |
| 6 | N(13)-C(14) | 1.2787 | 1.2600 | N(3)-N(13) | 1.4510 | 1.4260 |
| 7 | N(3)-N(13) | 2.0805 | 1.9260 | C(4)-O(11) | 1.3199 | 1.2080 |
| 8 | C(4)-O(11) | 1.2347 | 1.2080 | C(41)-O(50) | 1.3551 | 1.3550 |
| 9 | C(29)-O(36) | 1.2193 | 1.2080 | C(45)-C(46) | 1.4200 | 1.4200 |
| 10 | N(13)-Zn(26) | 1.9260 |  | N(13)-Cd(26) | 2.1560 |  |
| 11 | O(11)-Zn(26) | 1.8900 |  | O(11)-Cd(26) | 2.1200 |  |
| 12 | Zn(26)-Cl(27) | 2.2400 |  | Cd(26)-Cl(27) | 2.4700 |  |
| 13 | O(25)-Zn(26) | 1.8900 |  | O(25)-Cd(26) | 2.1200 |  |
| 14 | C(8)-H(30) | 1.1000 | 1.1000 | N(1)-C(10) | 1.2600 | 1.4560 |
| 15 | C(7)-H(29) | 1.1000 | 1.1000 | C(9)-C(10) | 1.3370 | 1.4200 |

**TABLE S5:** Some selected bond angles of optimized Cu(II) and Ni(II) complexes.

| Bond angles (°) | | | | | | |
| --- | --- | --- | --- | --- | --- | --- |
| No | Bonded atoms | [Cu(C_20_H_14_N_3_O_2_)_2_] | | Bonded atoms | [Ni(C_20_H_14_N_3_O_2_)_2_] | |
|  |  | Actual | Optimized |  | Actual | Optimized |
| 1 | C(44)-C(49)-C(40) | 89.0000 | 89.0100 | O(36)-Ni(51)-N(38) | 72.5347 |  |
| 2 | Cu(51)-N(13)-N(3) | 92.6670 |  | O(36)-Ni(51)-N(13) | 176.993 |  |
| 3 | Cu(51)-N(13)-C(14) | 91.2800 |  | N(38)-Ni(51)-O(11) | 90.3586 |  |
| 4 | N(3)-N(13)-C(14) | 94.2564 |  | N(3)-N(13)-C(14) | 95.2000 | 95.5400 |
| 5 | Cu(51)-O(11)-C(4) | 178.5400 |  | O(11)-Ni(51)-O(25) | 81.9421 |  |
| 6 | O(11)-Cu(51)-O(25) | 87.7421 |  | O(11)-C(4)-N(3) | 91.7992 | 91.6880 |
| 7 | O(11)-C(4)-N(3) | 90.1992 |  | N(13)-N(3)-C(4) | 89.2660 | 90.0000 |
| 8 | N(13)-N(3)-C(4) | 90.3338 |  | O(25)-C(16)-C(15) | 90.8391 | 91.0000 |
| 9 | O(25)-C(16)-C(15) | 88.9190 |  | Ni(51)-N(13)-N(3) | 91.7964 | 91.5800 |
| 10 | O(36)-Cu(51)-O(11) | 94.4700 |  | O(36)-Ni(51)-N(13) | 178.9935 |  |
| 11 | O(36)-Cu(51)-N(13) | 176.5400 |  | Ni(51)-N(38)-C(39) | 181.3290 | 181.1000 |
| 12 | O(36)-C(29)-C(30) | 171.4798 | 171.2400 | H(61)-C(18)-C(17) | 180.0607 | 180.0400 |
| 13 | H(61)-C(18)-C(17) | 180.0607 | 180.3200 | H(58)-C(12)-H(56) | 109.4618 | 109.0000 |
| 14 | H(58)-C(12)-H(56) | 174.4618 | 174.0254 | H(61)-C(18)-C(17) | 177.8003 | 177.6700 |
| 15 | C(4)-N(3)-C(2) | 86.9986 | 86.2846 | C(6)-C(5)-C(4) | 89.2692 | 89.3000 |

**TABLE S6:** Some selected bond angles of optimized Co(II) and Mn(II) complexes.

| Bond angles (°) | | | | | | |
| --- | --- | --- | --- | --- | --- | --- |
| No | Bonded atoms | [Co(C_20_H_14_N_3_O_2_)_2_] | | Bonded atoms | [Mn(C_20_H_14_N_3_O_2_)_2_] | |
|  |  | Actual | Optimized |  | Actual | Optimized |
| 1 | N(38)-Co(51)-O(11) | 93.8666 |  | Mn(51)-N(13)-N(3) | 93.7750 |  |
| 2 | O(36)-Co(51)-N(13) | 179.0214 |  | Mn(51)-N(13)-C(14) | 106.3752 |  |
| 3 | N(38)-Co(51)-O(11) | 93.8666 |  | N(13)-Mn(51)-O(50) | 83.2834 |  |
| 4 | N(3)-N(13)-C(14) | 95.0000 | 94.8000 | O(11)-Mn(51)-O(25) | 83.4727 |  |
| 5 | O(11)-Co(51)-O(50) | 93.6975 |  | O(25)-Mn(51)-O(50) | 164.7141 |  |
| 6 | O(11)-C(4)-N(3) | 95.9992 | 95.6000 | O(11)-C(4)-N(3) | 93.2066 | 193.2000 |
| 7 | N(13)-N(3)-C(4) | 88.2660 |  | N(13)-N(3)-C(4) | 92.0465 |  |
| 8 | O(25)-C(16)-C(15) | 98.9355 | 98.7000 | O(25)-C(16)-C(15) | 90.5362 | 90.5300 |
| 9 | Co(51)-N(13)-N(3) | 92.7964 | 92.0000 | Ni(51)-N(13)-N(3) | 90.7964 | 90.8000 |
| 10 | O(36)-Co(51)-N(13) | 179.5000 |  | O(36)-Mn(51)-O(50) | 181.2738 |  |
| 11 | Co(51)-N(13)-C(14) | 92.0000 | 92.0150 | Ni(51)-N(38)-C(39) | 182.3290 | 182.2000 |
| 12 | Co(51)-O(11)-C(4) | 180.0200 | 180.1000 | H(61)-C(18)-C(17) | 176.0607 | 176.0700 |
| 13 | O(25)-Co(51)-O(50) | 174.2081 |  | C(5)-C(10)-N(1) | 92.5758 | 92.4280 |
| 14 | C(10)-C(5)-C(6) | 88.9987 | 88.0500 | H(61)-C(18)-C(17) | 173.8003 | 173.7800 |
| 15 | C(4)-N(3)-C(2) | 91.9299 | 91.7200 | C(6)-C(5)-C(4) | 87.4251 | 87.6000 |

**TABLE S7:** Some selected bond angles of optimized Zn(II) and Cd(II) complexes.

| Bond angles (°) | | | | | | |
| --- | --- | --- | --- | --- | --- | --- |
| No | Bonded atoms | [Zn(C_20_H_14_N_3_O_2_)Cl] | | Bonded atoms | [Cd(C_20_H_14_N_3_O_2_)Cl] | |
|  |  | Actual | Optimized |  | Actual | Optimized |
| 1 | N(13)-Zn(26)-O(11) | 108.6726 |  | N(13)-Cd(26)-O(11) | 108.5704 |  |
| 2 | O(11)-Zn(26)-Cl(27) | 110.3896 |  | N(13)-Cd(26)-O(25) | 106.8074 |  |
| 3 | N(13)-Zn(26)-O(25) | 108.6156 |  | O(11)-Cd(26)-Cl(27) | 107.4474 |  |
| 4 | N(3)-N(13)-C(14) | 115.0000 | 120.0000 | N(3)-N(13)-C(14) | 107.8895 |  |
| 5 | Zn(26)-O(25)-C(16) | 108.4028 |  | Cd(26)-O(25)-C(16) | 107.2128 |  |
| 6 | O(11)-Zn(26)-O(25) | 113.1852 |  | O(11)-C(4)-N(3) | 105.1739 | 122.6000 |
| 7 | O(11)-C(4)-N(3) | 104.0450 | 122.6000 | N(13)-N(3)-C(4) | 101.2660 |  |
| 8 | N(13)-N(3)-C(4) | 110.5954 |  | O(25)-C(16)-C(15) | 112.4527 | 112.3000 |
| 9 | O(25)-C(16)-C(15) | 111.2161 | 111.0100 | Cd(26)-N(13)-N(3) | 105.6248 |  |
| 10 | Zn(26)-N(13)-N(3) | 106.6319 |  | O(36)-Cd(51)-N(13) | 109.5000 |  |
| 11 | O(36)-Zn(51)-N(13) | 109.5635 |  | Cd(26)-N(13)-N(3) | 105.6248 |  |
| 12 | H(33)-C(12)-H(32) | 108.3891 | 109.0000 | H(28)-C(6)-C(7) | 110.4908 | 110.0000 |
| 13 | H(58)-C(12)-H(56) | 109.4618 | 109.0000 | C(14)-C(15)-C(24) | 119.5837 | 120.0000 |
| 14 | H(28)-C(6)-C(5) | 110.8234 | 110.7000 | C(10)-C(5)-C(6) | 109.9987 | 109.8000 |
| 15 | C(4)-N(3)-C(2) | 108.3783 | 108.2000 | C(4)-N(3)-C(2) | 107.4999 | 107.8700 |
